# Biological characteristics of pregnancy in captive Yangtze finless porpoises revealed by urinary metabolomics^†^

**DOI:** 10.1093/biolre/ioad175

**Published:** 2024-01-03

**Authors:** Bin Tang, Yujiang Hao, Chaoqun Wang, Zhengyu Deng, Zhangbing Kou, Haojie Zhou, Haobo Zhang, Fei Fan, Kexiong Wang, Ding Wang

**Affiliations:** Key Laboratory of Aquatic Biodiversity and Conservation, Institute of Hydrobiology, Chinese Academy of Sciences, Wuhan, China; Key Laboratory of Aquatic Biodiversity and Conservation, Institute of Hydrobiology, Chinese Academy of Sciences, Wuhan, China; National Aquatic Biological Resource Center, NABRC, Wuhan, China; Key Laboratory of Aquatic Biodiversity and Conservation, Institute of Hydrobiology, Chinese Academy of Sciences, Wuhan, China; National Aquatic Biological Resource Center, NABRC, Wuhan, China; Key Laboratory of Aquatic Biodiversity and Conservation, Institute of Hydrobiology, Chinese Academy of Sciences, Wuhan, China; National Aquatic Biological Resource Center, NABRC, Wuhan, China; Key Laboratory of Aquatic Biodiversity and Conservation, Institute of Hydrobiology, Chinese Academy of Sciences, Wuhan, China; Key Laboratory of Aquatic Biodiversity and Conservation, Institute of Hydrobiology, Chinese Academy of Sciences, Wuhan, China; University of Chinese Academy of Sciences, Beijing, China; Key Laboratory of Aquatic Biodiversity and Conservation, Institute of Hydrobiology, Chinese Academy of Sciences, Wuhan, China; University of Chinese Academy of Sciences, Beijing, China; Key Laboratory of Aquatic Biodiversity and Conservation, Institute of Hydrobiology, Chinese Academy of Sciences, Wuhan, China; National Aquatic Biological Resource Center, NABRC, Wuhan, China; Key Laboratory of Aquatic Biodiversity and Conservation, Institute of Hydrobiology, Chinese Academy of Sciences, Wuhan, China; National Aquatic Biological Resource Center, NABRC, Wuhan, China; Key Laboratory of Aquatic Biodiversity and Conservation, Institute of Hydrobiology, Chinese Academy of Sciences, Wuhan, China; National Aquatic Biological Resource Center, NABRC, Wuhan, China

**Keywords:** Yangtze finless porpoises, urinary metabolomics, pregnancy, biomarker

## Abstract

The Yangtze finless porpoises (*Neophocaena asiaeorientalis a.*) are an endemic and critically endangered species in China. Intensive captive breeding is essential for understanding the biology of critically endangered species, especially their pregnancy characteristics, knowledge of which is crucial for effective breeding management. Urine metabolomics can reveal metabolic differences, arising from physiological changes across pregnancy stages. Therefore, we used the urinary metabolomic technology, to explore urinary metabolite changes in pregnant Yangtze finless porpoises. A total of 2281 metabolites were identified in all samples, which including organic acids and derivatives (24.45%), organoheterocyclic compounds (20.23%), benzenoids (18.05%), organic oxygen compounds (7.73%), and phenylpropanoids and polyketides (6.48%). There were 164, 387, and 522 metabolites demonstrating differential abundance during early pregnancy, mid pregnancy, and late pregnancy, respectively, from the levels observed in nonpregnancy. The levels of pregnenolone, 17α-hydroxyprogesterone, and tetrahydrocortisone were significantly higher during all pregnancy stages, indicating their important roles in fetal development. The differential metabolites between nonpregnancy and pregnancy were mainly associated with amino acid and carbohydrate metabolism. Moreover, metabolic activity varied across pregnancy stages; steroid hormone biosynthesis was predominant in early pregnancy, and amino acid biosynthesis and carbohydrate metabolism were predominant in mid pregnancy and late pregnancy, respectively. Our results provide new insights into metabolic characteristics in the Yangtze finless porpoises’ urine during pregnancy, and indicate that the differential levels of urine metabolites can determine pregnancy in Yangtze finless porpoises, providing valuable information for the husbandry and management of pregnant Yangtze finless porpoises in captivity.

## Teaser

Pregnenolone, 17α-hydroxyprogesterone, and tetrahydrocortisone play a crucial role in the pregnancy of Yangtze finless porpoises and can serve as biomarkers for pregnancy management.

## Graphical Abstract

**Graphical Abstract:**
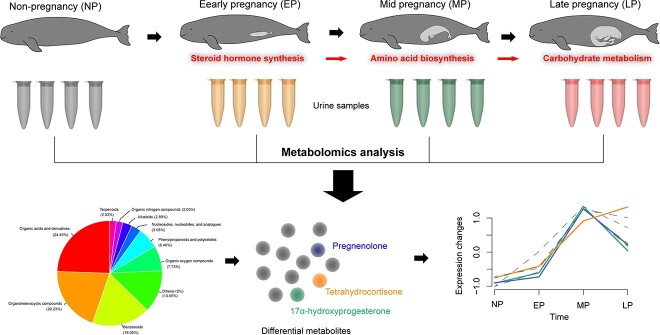

## Introduction

Cetaceans are aquatic mammals requiring long gestation periods to reproduce offspring [1, 2]. Guaranteeing effective protection of cetaceans in the wild requires good knowledge of their reproductive physiology [3–5]. However, obtaining such knowledge about cetaceans is challenging due to the limitations of their aquatic environment [5].

Pregnancy in mammals is generally divided into three trimesters, each lasting approximately one-third of the total pregnancy [6–8]. Several studies of terrestrial mammals have revealed fetal development characteristics at different trimesters of pregnancy. During the first trimester, the endometrium of the uterus forms a trophoblast, where the embryo implants in the uterus and begins to differentiate into various tissues and organs. These sudden changes can trigger immune rejection reactions in the maternal body, causing physiological changes in the mother, including nausea, fatigue, breast tenderness, and frequent urination [9–11]. Most miscarriages and congenital abnormalities occur during this period [10, 12, 13]. In the second trimester, the early body systems and structures established during the embryonic stage continue to develop, and fetal growth and weight continue to increase. During the third trimester, the fetus grows rapidly, and its weight increases significantly [14–17]. Fetal development in cetaceans is similar to that in terrestrial mammals, and the pregnancy process can be divided into three stages according to variations in steroid hormones [18, 19]. However, ultrasonic examination of cetacean fetuses revealed that, unlike humans, these species do not show a significant trend of rapid growth in the late pregnancy stage [19–22].

Pregnancy is associated with various adaptive processes that change during gestation to accommodate fetal development. It is well documented that maternal steroid hormone levels undergo substantial variation during pregnancy in nearly all mammalian species [18, 23–25]. These hormonal fluctuations are crucial for regulating various physiological processes and ensuring the successful progression of pregnancy [24, 26]. Furthermore, studies have shown that pregnancy impacts amino acid metabolism in various mammalian species. For example, research on pandas (*Ailuropoda melanoleuca*), rats, and pigs has demonstrated alterations in amino acid utilization and metabolism during pregnancy [27–31]. These changes reflect the increased demand for nutrients and the shifting metabolic priorities that support fetal growth and development [32]. In addition to hormonal and metabolic changes, pregnancy significantly influences the immune response. The immune system undergoes dynamic modifications to establish immune tolerance toward the developing fetus while maintaining the mother’s ability to protect against pathogens [33, 34]. These immunological adaptations help prevent immune rejection of the fetus, ensuring its survival and normal development.

Research on the physiological changes during pregnancy in cetacean species has primarily focused on hormone fluctuation patterns, particularly in captive individuals [18, 35–38]. However, further in-depth investigations are needed to unravel the full extent of maternal physiological changes during pregnancy in cetaceans. By conducting more comprehensive studies, we can better understand the physiological adaptations that occur during the gestation period in cetaceans. This knowledge will be crucial for effectively managing and caring for pregnant cetaceans, ensuring their well-being and successful parturition.

Metabolomics is the study of metabolites and their chemical processes in biological samples. It encompasses the analysis of various small molecules present in cells, tissues, and biofluids, providing valuable insights into metabolic pathways and their regulation [39]. Mammalian urine, in particular, offers several advantages as a metabolomic analysis sample. It contains a wide range of metabolites, making it a rich source of metabolic information [40]. Furthermore, urine can be collected noninvasively, minimizing any potential harm or stress to the animals [41]. As a humoral circulation metabolite, urine provides a comprehensive representation of the biochemical pathways occurring in the body [40, 42]. Therefore, urine-based metabolomics is a valuable tool for studying metabolic changes and understanding physiological processes in mammals.

This study aimed to investigate the urinary metabolites of pregnant Yangtze finless porpoises (YFPs; *Neophocaena asiaeorientalis a.*) to reveal physiological changes during pregnancy and identify potential urinary metabolites as pregnancy biomarkers. The YFP is a critically endangered freshwater cetacean species [43], and examining the metabolic profiles of this species helps fill the knowledge gap regarding its physiological adaptations during pregnancy. Moreover, identifying specific urinary metabolites associated with pregnancy facilitates the development of artificial insemination techniques for this species, ultimately contributing to YFP conservation and management efforts. The findings of this study provide valuable insights and serve as a reference for reproductive biology research in cetaceans.

## Methods

### Study animals and sample collection

In this study, two sexually mature female captive YFPs, YY and F9, were investigated. These animals were originally wild-caught from the Poyang Lake at approximately 2 years of age in 2009 and 2011, respectively, and were subsequently housed in the Baiji Dolphinarium (Wuhan, China). The animals were mainly fed crucian carp (*Carassius auratus*), common carp (*Cyprinus carpio*), and sharpbelly (*Hemiculter leucisculus*), and the daily intake was adjusted based on the animals’ seasonal changes in body mass and appetite (approximately 6–8% of their body weight per day). All the animals shared the same water-sustaining system, and the water temperature changed seasonally, ranging from 11°C to 27°C. From 2017 to 2022, the two YFPs had four successful reproductive events, one for YY and three for F9 (Supplementary Table S1). Reproductive success was defined as the delivery of a calf that survived ≥30 days [19].

Urine samples were collected from the captive YFPs during the morning feeding process following the guidelines for urine sample collection described by Hao et al. [37]. The urine samples were collected voluntarily with operant conditioning. Briefly, the animal was asked to turn their belly up under the behavioral stimulus of the trainers. Then, the trainer gently dabbed the genital area of the animal with their fingers. If the animal urinated, the trainer collected midstream urine with a spoon and immediately reinforced the behavior. Then, the urine sample was transferred into a centrifuge tube and labeled with the animal’s ID and date. The collected urine samples were immediately frozen and stored at −80°C for metabolite investigation.

The average gestation of YFPs was estimated to be 349 ± 18 days, which is nearly 1 year [20]. Hormone studies of YFPs and bottlenose dolphins (with gestation similar to that of YFPs) have revealed that the changes in progesterone and testosterone levels during pregnancy can be roughly divided into three stages: the first 4 months represent the rising period, the subsequent 4 months indicate the stable period, and the last 4 months signify the declining period [18, 44, 45]. Most studies adopt these three corresponding stages to categorize pregnancy. Thus, the YFP gestation in this study was divided into three stages as follows: late pregnancy (LP) (about the 4 months before parturition), mid pregnancy (MP) (about the mid 4 months of pregnancy), and early pregnancy (EP) (the early 4 months of gestation). The urinary samples (*n* = 16) used in this study were collected across all gestation stages, with one sample selected from each gestation stages of the four pregnancy events. Four urinary samples collected before conception per pregnancy event were used as the control group (NP) (Supplementary Table S1).

The study methods, including animal training and urine sample collection, were approved by the Research Ethics Committee of the Institute of Hydrobiology, Chinese Academy of Sciences. The study was conducted in strict accordance with Chinese laws and ethical guidelines for wildlife.

### Metabolite extraction and quality control

Urinary samples were thawed slowly at 4°C, and then 100 μL of the urinary sample was placed in a 96-well plate. Three hundred microliters of an extraction solution (methanol: acetonitrile = 2:1, v:v, precooled at −20°C) was added to extract the metabolites. The mixture was vortexed for 1 min and kept at −20°C for 2 h. Subsequently, the mixed solution was centrifuged at 4000 × rcf for 30 min at 4°C and 300 μL of the supernatant was collected and dried using a freezing vacuum concentrator. Then, they were reconstituted with 150 μL methanol: water (1,1, v,v) reconstitution solution. After vortexing for 1 min, the samples were centrifuged at 4000 × rcf for 30 min at 4°C. The resulting supernatant was used for liquid chromatography–mass spectrometry (LC–MS) analysis.

To evaluate the stability and repeatability of the LC–MS analysis process, 10 μL of the supernatant from each sample was mixed to produce the quality control (QC) sample [46]. The samples were randomly sorted during instrumental analysis to reduce systematic errors, and a QC sample was assessed every 10 samples.

### Liquid chromatography–mass spectrometry analysis conditions

Chromatographic separation was performed using a Waters 2D UPLC (Waters, USA) system equipped with a BEH C18 column (1.7 μm, 2.1 × 100 mm, Waters, USA). The mobile phase of the positive-ion mode consisted of 0.1% formic acid in water (solvent A) and 0.1% formic acid in methanol (solvent B). The mobile phase of the negative-ion mode consisted of 10 mM ammonium formate in water (solvent A) and 10 mM ammonium formate in 95% methanol (solvent B). The gradient elution conditions were as follows: 2% solvent B for 0–1 min; 2–98% solvent B for 1–9 min; 98% solvent B for 9–12 min; 98–2% solvent B for 12–12.1 min; and 2% solvent B for 12.1–15 min. The sample injection volume was 5 μL at a flow rate of 0.35 mL/min. The column temperature was maintained at 45°C.

A Q Exactive HF mass spectrometer (Thermo Fisher Scientific, USA) was used to collect primary and secondary mass spectra data. Detection was performed in the mass range of 70–1050 m/z, the primary resolution was 120 000, the AGC was 3e6, and the maximum injection time (IT) was 100 ms. According to the parent ion power, Top3 was selected for fragmentation; then, secondary mass spectra data were collected with a resolution of 30 000. The AGC was 1e5, the maximum IT was 50 ms, and the stepped normalized collision energy (NCE) was set to 20, 40 and 60 eV. The electrospray ionization (ESI) source conditions were as follows: a sheath gas flow rate of 40, aux gas flow rate of 10, and a spray voltage of 3.80 kV for the positive-ion mode and 3.20 kV for the negative-ion mode. The capillary temperature was 320°C, and the aux gas heater temperature was 350°C.

### Metabolomics data processing

The raw data collected by LC–MS/MS were imported into Compound Discoverer 3.1 (Thermo Fisher Scientific, USA) for data processing [47, 48], including peak picking, retention time correction, adduct ion coalescing, missing data filling, background peak labeling, and metabolite identification. The processing result comprises the compound molecular weight, retention time, peak area, and identification result. Metabolite identification was coupled with the BMDB, mzCloud, ChemSpider, HMDB, KEGG, and LipidMaps databases. The main parameters for metabolite identification were precursor mass tolerance <5 ppm, fragment mass tolerance <10 ppm, and retention time tolerance <0.2 min.

The peak area data were further preprocessed using metaX. First, probabilistic quotient normalization (PQN) was used to normalize the data and calculate the relative peak area. Then, QC-based robust LOESS signal correction (QC–RLSC) was used to correct the batch effects [46, 49]. Finally, compounds with a relative peak area coefficient of variation (CV) >30% were removed from the QC samples.

### Statistical analysis

Multivariate statistical analyses, including principal component analysis (PCA) and orthogonal partial least square–discriminant analysis (OPLS–DA), were performed using SIMCA 14.1 software (Umetrics, Sweden) [50]. Principal component analysis using an unsupervised method was performed to obtain an overview of the metabolic data. OPLS–DA was performed to further explore the metabolites that contributed most to the differences across pregnancy periods. The variable importance in projection (VIP) obtained from the OPLS–DA model, combined with the fold change analysis and univariate Student *t-*test (*p* < 0.05), was used to select important differential metabolites. Differential metabolites were screened using the following criteria: VIP ≥ 1; ratio ≥ 2 or ratio ≤ 0.5；*p* < 0.05. The top 30 different metabolites were selected in the metabolic concentration of each pregnancy period, relative to the nonpregnancy period.

Different metabolite trend analysis was performed using Short Time-series Expression Miner software (STEM) on the OmicShare tools platform, a free online platform for data analysis (www.omicshare.com/tools) [51]. In addition, pathway analysis was performed with MetaAnalyst 5.0, a web-based tool for visualization of metabolomics (www.metaboanalyst.ca/MetaboAnalyst). Various differential metabolites between the pregnancy and nonpregnancy periods were summarized and mapped to their biochemical pathways through metabolic enrichment and pathway analysis based on the Kyoto Encyclopedia of Genes and Genomes (KEGG) database.

## Results

### Untargeted metabolic profiling of urine during pregnancy

Nontargeted metabolomics analysis was performed to explore metabolic changes in urine during the pregnancy of captive YFPs. The QC results showed that our experiment had essential repeatability and stability through the analytical run (Supplementary Figure S1A and B). A total of 4788 and 2015 features were extracted from the positive-ion and negative-ion modes, respectively. After normalization, 4258 and 1573 features with a relative standard deviation (RSD) of <30% accounted for 88.93% and 78.06% of all QC samples, respectively. A total of 2281 annotated metabolites were identified, with 1605 metabolites detected in the positive-ion mode and 676 metabolites detected in the negative-ion mode (Supplementary Table S2).

**Figure 1:**
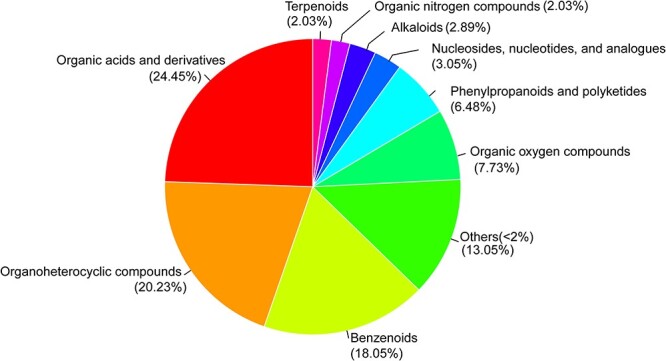
The pie chart of metabolite classification according to category information (Super class) from the HMDB 5.0 database. The different colors represent different HMDB categories. The metabolites with percentages lower than 2% were clustered into “other,” accounting for 13.05%.

The taxonomic information of metabolites obtained from the HMDB 5.0 database showed that all the identified metabolites could be classified into 39 metabolic categories (Super class) (Supplementary Table S3). The top five metabolic categories were organic acids and derivatives (24.45%), organoheterocyclic compounds (20.23%), benzenoids (18.05%), organic oxygen compounds (7.73%), and phenylpropanoids and polyketides (6.48%), which accounted for 77.39% of the total metabolites (Figure 1).

### Potential metabolic biomarkers for pregnant Yangtze finless porpoises

**Figure 2:**
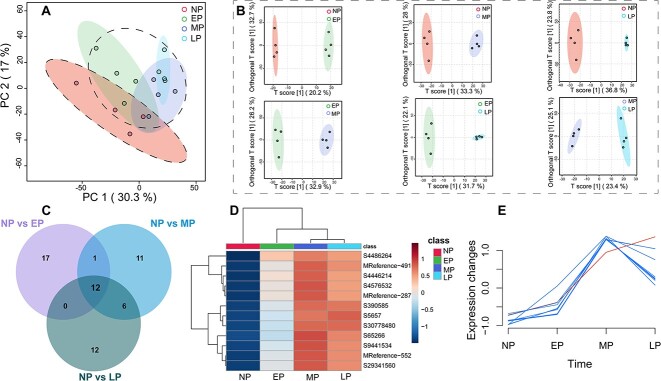
Differentially abundant metabolite selection process. NP, nonpregnancy stage; EP, early pregnancy stage; MP, mid pregnancy stage; LP, late pregnancy stage. (A) PCA score plot shows the difference between the pregnancy and nonpregnancy groups. (B) OPLS–DA further revealed the differences among the comparable groups. (C) Overlap of metabolites between pregnancy periods (EP, MP, and LP) and the nonpregnancy period (NP). (D) Heatmap of the relative intensity of 12 potential biomarkers with pregnancy progression. (E) Time expression features of 12 potential biomarkers. Tetrahydrocortisone (ID: S5657), showed a different expression feature compared to the other metabolites, with increased levels in late pregnancy.

Principal component analysis was performed to explore the metabolic changes during pregnancy progression. The PCA score plot showed obvious differences between the metabolites in the pregnant and nonpregnant periods. Moreover, the EP metabolites differed from those in LP, while MP metabolites overlapped those in EP and LP (Figure 2A). The OPLS–DA score plot showed apparent separations between the NP and the three pregnancy groups (NP vs. EP, NP vs. MP, and NP vs. LP) and between each pregnancy group (EP vs. MP, EP vs. LP, and MP vs. LP) (Figure 2B). Permutation tests with 200 iterations were performed to validate the OPLS–DA models, indicating no overfitting (Supplementary Figure S2).

The differential metabolites that satisfied the criteria of VIP ≥ 1, ratio ≥ 2 or ratio ≤ 0.5 and *p* < 0.05 were considered biomarker candidates. Hence, 164, 387, and 522 differential metabolites were identified from the comparisons of EP vs. NP, MP vs. NP, and LP vs. NP, respectively (Supplementary Table S4). Moreover, 402, 395, and 112 differential metabolites were identified from the comparisons of EP vs. MP, EP vs. LP, and MP vs. LP, respectively (Supplementary Table S4). Furthermore, the top 30 differential metabolites at three pregnancy stages compared to the nonpregnancy stage were selected for further analysis to screen the potential metabolic biomarkers for pregnant YFPs from a wide range of differential metabolites. A Venn diagram was constructed to depict 12 differential metabolites overlapping three comparisons between the pregnancy and nonpregnancy periods (NP vs. EP, NP vs. MP, and NP vs. LP), namely, (+/−)-CP 47,497-C7-hydroxy metabolite, sunitinib, pregnenolone (P5), N-oleoyl-l-serine, trandolaprilat, (5Z,9α,11α,13E,15S)-11-acetoxy-9,15-dihydroxyprosta-5,13-dien-1-oic acid, cardanol triene, 17α-hydroxyprogesterone (17-OHP), grayanotoxin I, nomilin, tetrahydrocortisone (THE), and N-phenylacetyl pyroglutamic acid, which could serve as potential metabolic biomarkers for pregnant YFPs (Figure 2C).

The relative levels of these 12 potential metabolic biomarkers are presented as a heatmap, showing consistent upregulated in pregnancy periods (Figure 2D). Furthermore, the time-series analysis showed a similar time expression feature of 12 potential metabolic biomarkers during pregnancy progression. All 12 potential metabolic biomarkers increased from NP to MP and increased rapidly from EP to MP. After MP, 11 of 12 potential metabolic biomarkers began to decrease, and THE continued to increase (Figure 2E).

### Metabolic pathways for the differential metabolites

**Figure 3:**
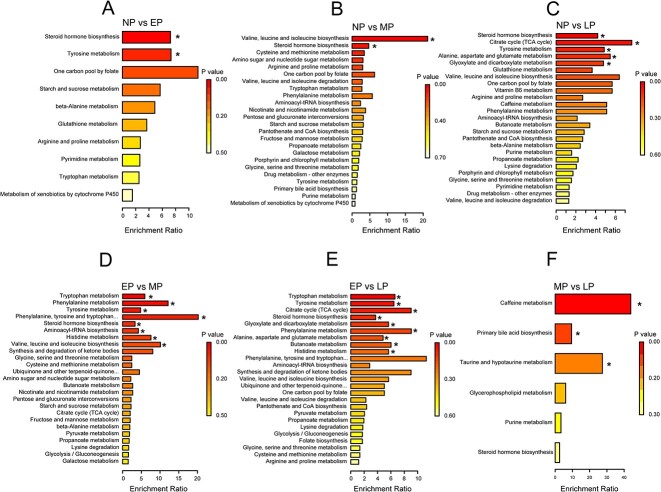
Pathway analysis summary histogram. Differential metabolites were classified by KEGG pathway enrichment and significance analysis. Histograms A, B, C, D, E, and F represent the comparisons of NP vs. EP, NP vs. MP, NP vs. LP, EP vs. MP, EP vs. LP, and MP vs. LP, respectively.

Metabolite set enrichment analysis was performed on the differential metabolites using MetabAnalyst 5.0 to identify dysregulated metabolic pathways. The results showed that the biological functions of differential metabolites were primarily associated with amino acid metabolism and carbohydrate metabolism (Figure 3A–C; Supplementary Table S5). Notably, steroid hormone biosynthesis was the only co-enriched pathway in each comparison (Figure 3, Supplementary Table S5).

However, there were obvious differences in functional enrichment across the pregnancy periods. Compared with the nonpregnancy periods, in the EP periods, the differential metabolites were enriched in 10 metabolic pathways, of which steroid hormone biosynthesis and tyrosine metabolism were the significantly enriched pathways (*p* < 0.05, Figure 3A). Compared with the nonpregnancy periods, in the MP periods, the differential metabolites were enriched in 24 metabolic pathways, of which valine, leucine, and isoleucine biosynthesis and steroid hormone biosynthesis were the significantly enriched pathways (*p* < 0.05, Figure 3B). Compared with the nonpregnancy periods, in LP periods, the differential metabolites were enriched in 27 metabolic pathways, and the significantly enriched pathways, in order, were steroid hormone biosynthesis; citrate cycle (TCA cycle); tyrosine metabolism; alanine, aspartate, and glutamate metabolism; and glyoxylate and dicarboxylate metabolism (*p* < 0.05, Figure 3C).

Furthermore, functional enrichment analysis was performed on the differential metabolites from the comparison of EP vs. MP, EP vs. LP, and MP vs. LP to investigate functional differences during pregnancy. The differential metabolites from EP vs. MP comparison were enriched in 36 metabolic pathways, of which tryptophan metabolism was the most enriched pathway, and the other metabolic pathways were mainly associated with amino acid metabolism and carbohydrate metabolism (Figure 3D, Supplementary Table S5). The differential metabolites from the EP vs. LP comparison were enriched in 27 metabolic pathways, similar to the comparison of EP vs. MP, and the metabolic pathways were mainly associated with amino acid metabolism and carbohydrate metabolism (Figure 3E, Supplementary Table S5). The differential metabolites from the MP vs. LP comparison were enriched in six metabolic pathways, of which caffeine metabolism was the most enriched pathway, and the other metabolic pathways were mainly associated with lipid metabolism (Figure 3F, Supplementary Table S5).

## Discussion

Metabolomics has emerged as a powerful approach in prenatal research for mammals, including humans, as it provides detailed insights into the pregnancy process and can identify potential diagnostic or predictive purposes [23, 27, 28, 52, 53]. In this study, we comprehensively characterized the maternal urine metabolome of two captive YFPs across four pregnancy events. The results revealed that the urine metabolites of YFPs were similar to those observed in other mammals, with a predominant organic acids and derivatives and organoheterocyclic compounds [27, 28, 40, 54]. These findings provide valuable baseline data on the urine metabolome of YFPs and may contribute to future research on pregnancy in marine mammals.

Pregnancy is associated with the onset of many physiological adaptation processes that are likely to change over the course of gestation to adapt to fetal development [24, 53, 55, 56]. In the present study, we investigated longitudinal variation in the urinary metabolome during pregnancy in YFPs and explored potential biomarkers for pregnancy prediction. Through multivariate analysis, we identified 12 metabolites that exhibited significant increases during the three stages of pregnancy that are considered candidate biomarkers for pregnancy in YFPs. Importantly, three of these metabolites, P5, 17-OHP, and THE, were found to be associated with steroid hormone metabolism (Figure 2). The other nine metabolites lack sufficient research evidence to establish their biological significance. Therefore, further in-depth investigations are necessary to determine their roles and potential relevance in pregnancy processes.

P5 is a naturally occurring steroid hormone that serves as a precursor or metabolic intermediate in the biosynthesis of several steroid hormones, including progestogens, androgens, estrogens, glucocorticoids, and mineralocorticoids [57]. Moreover, P5 possesses its own biological activity and functions as a neurosteroid, influencing neurotransmitters activity [58, 59]. During pregnancy, P5, a precursor of progesterone, plays a crucial role in the female body and can also be transported to the fetus through the bloodstream and affect fetal neurodevelopment, organ development, and immune system maturation [24, 59, 60]. Insufficient P5 may hinder progesterone synthesis, potentially leading to miscarriage, preterm birth, fetal growth restriction, and preeclampsia [61, 62]. The primary source of P5 is its synthesis through the metabolic and cleavage processes of cholesterol [63]. Therefore, proper regulation of cholesterol is essential for a healthy pregnancy.

17α-Hydroxyprogesterone is an endogenous progestogen produced during the synthesis of glucocorticoids and sex steroids, and it can be converted from progesterone by 17α-hydroxylase or from P5 by 17α-hydroxyprogesterone deacetylase and 3β-hydroxysteroid dehydrogenase [63–65]. 17α-Hydroxyprogesterone mainly promotes organ development in conjunction with sex hormones [64, 66]. During pregnancy, the fetus, placenta, and adrenal gland can produce a large amount of 17α-OHP [67]. In the present study, we found significantly higher levels of 17-OHP in the urine of YFPs during pregnancy, suggesting that it plays a crucial role in YFP fetal development.

Tetrahydrocortisone is a metabolite of the hormone cortisol. It is an inactive form of cortisol without substantial biological activity [68]. Tetrahydrocortisone is commonly measured in urine as a marker of cortisol production and metabolism [69]. In cetaceans, elevated concentrations of fecal glucocorticoids during pregnancy have been observed in the North Atlantic right whale (*Eubalaena glacialis*) [70], blue whale (*Balaenoptera musculus*) [38], humpback whales (*Megaptera novaeangliae*) [71], and YFPs [72]. Furthermore, long-term continuous monitoring by Steinman et al. [73] revealed that serum cortisol levels in bottlenose dolphins (*Tursiops truncatus*) showed a sustained increase during pregnancy, reaching a peak in the final month of gestation [73]. Previous research on terrestrial mammals has indicated a significant increase in glucocorticoid levels, including cortisol, during pregnancy. This increase is believed to result from the higher energy requirements of this period [74, 75] and is essential for the development and maturation of the respiratory system in late gestation [76, 77]. The continuous increase in urinary THE levels in this study may represent a similar trend in cortisol during pregnancy (Figure 2E). Additionally, the high food intake and thick blubber layer of pregnant YFPs further indicate that the increase in cortisol during pregnancy is due to the increased demand for energy metabolism [78].

Moreover, P5, 17-OHP, and THE are important metabolites in the steroid hormone biosynthesis pathway, which is the most significantly enriched pathway during YFP gestation (Figure 3). Numerous studies have highlighted the crucial role of steroid hormones in mammalian pregnancy, such as preparing for mammary gland secretion, regulating uterine activity, and promoting fetal organ development [55, 79–82]. In this metabolic pathway, P5 is a direct precursor in progesterone synthesis, 17-OHP is a direct metabolite of progesterone, and THE is the ultimate metabolite of progesterone [63]. In this study, we observed a continuous increase in P5 and 17-OHP levels from EP to MP, followed by a decline during LP (Figure 2E). This trend is consistent with the changes observed in progesterone levels in other pregnant cetaceans [83–85], suggesting that the urinary metabolic levels of P5 and 17-OHP can reflect the variations in progesterone levels. The decline in progesterone levels during the LP may indicate the initiation of parturition in YFPs. Progesterone levels exhibit two distinct patterns of decline in the later stages of pregnancy [86, 87]. One pattern involves the upregulation of 17α-hydroxylase expression, which facilitates the conversion of progesterone into estrogens, a process commonly referred to as “progesterone withdrawal,” as observed in animals such as sheep and horses [88, 89]. The other pattern is characterized by the absence of 17α-hydroxylase expression, resulting in relatively stable progesterone levels during the later stages of pregnancy. However, in this pattern, there is a decline in the binding capacity of progesterone receptors, a condition referred to as “functional progesterone withdrawal,” as observed in humans, guinea pigs, and primates [87, 90]. Yangtze finless porpoises belong to the former category, according to our results. However, previous research indicates that there is no significant increase in estrogen levels in YFPs and other cetaceans during the LP stage [83]. The upregulation of 17α-hydroxylase expression in cetaceans requires further investigation. In addition, P5 serves as the common precursor for the synthesis of steroid hormones and significantly decreases in the LP stage in YFPs (Figure 2E). This decline may be the primary factor contributing to the decrease in progesterone levels and the initiation of parturition. Furthermore, our study revealed that THE continues to increase during the LP stage, potentially reflecting changes in cortisol levels. Elevated cortisol levels can stimulate prostaglandin synthesis and initiate parturition, a process observed in most mammals. These findings may suggest a unique parturition initiation mechanism in cetaceans, possibly achieved by controlling the conversion of cholesterol into P5 to achieve progesterone withdrawal. Given the crucial roles of these three metabolites during gestation and the initiation of parturition, they can be considered potential signature metabolites for predicting pregnancy and parturition in YFPs and other small cetaceans.

The results of the differential metabolite set enrichment analysis indicate that during YFP pregnancy, in addition to the significant enrichment of steroid hormone biosynthesis, three amino acid metabolic pathways, namely, tyrosine metabolism; valine, leucine, and isoleucine biosynthesis; and alanine, aspartate, and glutamate metabolism, are significantly enriched (Figure 3). Amino acids play a crucial role in the growth, development, and health of the fetus while also providing vital support for the organs and systems of both the mother and the fetus during pregnancy [31]. Tyrosine plays a pre-regulatory role in the development of dopaminergic systems in the offspring after birth [91]. Glutamine serves as a substrate for protein synthesis, a promoter of muscle growth, and the primary metabolic fuel source for rapidly dividing cells in the placenta and fetal liver [92]. Valine, leucine, and isoleucine, collectively referred to as branched-chain amino acids (BCAAs), play a crucial role in regulating body protein mass and insulin growth factors [30]. Therefore, their presence is essential for fetal growth and a healthy pregnancy [93, 94]. Previous studies have also demonstrated that BCAA supplementation can enhance fetal growth and increase birth weight [30, 94]. Given the potential crucial roles of these amino acids in the pregnancy and fetal development of YFPs, it is recommended to provide an adequate supply during gestation.

Additionally, two carbohydrate metabolism pathways, the TCA cycle and glyoxylate and dicarboxylate metabolism, exhibited significant enrichment (Figure 3). These two pathways are essential aerobic pathways for the oxidation of carbohydrates and fatty acids, providing energy to the organism. Notably, energy expenditure increases during YFP gestation, a phenomenon shared with other mammals [31, 32].

The pathway analysis further indicated distinct physiological changes during each stage of YFP pregnancy (Figure 3). At the EP stage, the most significant changes are observed in steroid hormone biosynthesis, as these hormones play an important role in promoting endometrial development, embryo implantation, and maternal immune adaptation [24, 33, 95]. These changes effectively prevent the risk of miscarriage and congenital abnormalities during the early stages of pregnancy. At the MP stage, the most significant changes occurred in amino acid metabolism. This can be attributed to the rapid development of various fetal systems and organs requiring substantial protein synthesis [96, 97]. At the LP stage, in addition to changes in steroid hormone biosynthesis and amino acid metabolism, significant changes occurred in carbohydrate metabolism. Research has shown that the human fetus undergoes rapid development and experiences a significant increase in weight during the third trimester [14–17], while in cetaceans including the YFP, fetal growth follows a linear trend without a rapid growth stage [19–22]. Therefore, the significant changes in carbohydrate metabolism may function to provide sufficient energy for fetal growth but rather allow the mother to store sufficient energy for lactation.

The YFP is a critically endangered species with a population of only approximately 1000 individuals [43]. Currently, only two adult female animals are available for artificial breeding research, and successful breeding cases are limited. Therefore, this study was limited by a small sample size, which restricted our ability to explore the physiological mechanisms of YFP pregnancy in depth. Individual variations might obscure common patterns. Therefore, further data and research are needed. Additionally, the sampling intervals in this study were relatively coarse; thus, a more detailed experimental design is necessary to validate the reliability and effectiveness of the potential biomarkers identified (P5, 17-OHP, and THE). However, as one of the few studies investigating the pregnancy physiology of small cetaceans using urine samples, this research provides a new perspective on this topic and offers guidance for managing cetacean artificial breeding.

## Conclusion

Urinary metabolomics of YFPs during pregnancy was determined by LC–MS/MS. A total of 12 urinary metabolites were identified as candidate biomarkers for pregnancy in YFPs (Figure 2). In particular, P5, 17-OHP, and THE associated with steroid hormone biosynthesis play a critical role throughout the pregnancy process. This finding supports the evidence that steroid hormone biosynthesis is crucial in mammalian pregnancy. Pathway analysis revealed that the differential metabolites between the nonpregnancy and pregnancy group were mainly associated with amino acid metabolism and carbohydrate metabolism (Figure 3). Furthermore, notable variations in metabolite levels were observed across different stages of pregnancy, indicating changes in metabolic processes during pregnancy. Specifically, during the EP stage, the emphasis appeared to be on steroid hormone synthesis. In contrast, during the MP and LP stages, the metabolic focus shifted toward amino acid biosynthesis and carbohydrate metabolism, respectively. These findings enhance our understanding of metabolic dynamics during pregnancy and help to identify potential biomarkers for monitoring and predicting pregnancy and parturition in YFPs. Further research in this area is warranted to elucidate the intricate interactions and regulatory processes among the identified metabolites and to validate their reliability and effectiveness as biomarkers. Continued investigations will enhance our understanding of the complex metabolic pathways involved in YFP pregnancy and facilitate the development of targeted reproductive health monitoring and management strategies for this endangered species.

## Supplementary Material

Supplementary_figtures_ioad175

Table_S1_ioad175

Table_S2_ioad175

Table_S3_ioad175

Table_S4_ioad175

Table_S5_ioad175

## Data Availability

The data underlying this article are available in the article and in its online supplementary material.
