# Supplementary material for: Biological characteristics of pregnancy in captive Yangtze finless porpoises revealed by urinary metabolomics
Source: Biol Reprod. 2024 Jan 3;110(4):808–18. doi: 10.1093/biolre/ioad175 (PMC11017131; doi:10.1093/biolre/ioad175)
Supplement: Supplementary_figtures_ioad175 [file supplementary_figtures_ioad175.docx]

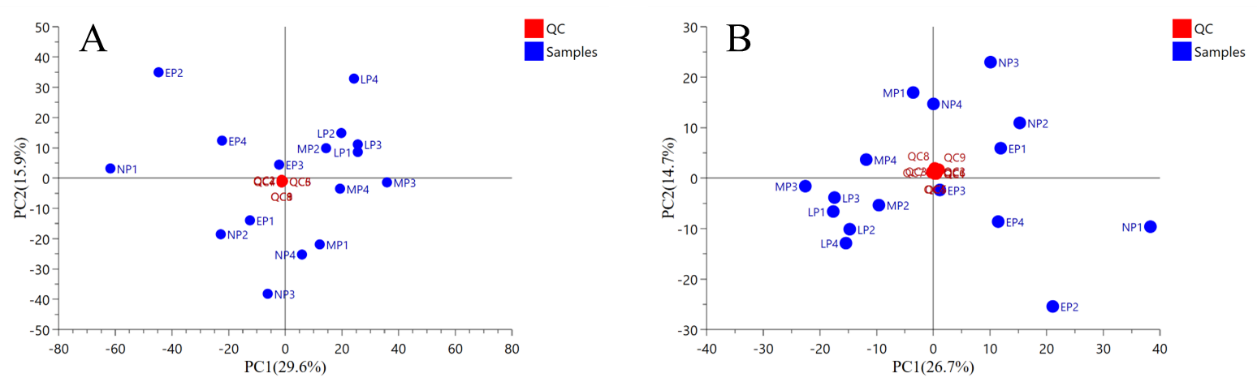


**Figure S1** Principal component analysis (PCA) score plots of metabolites profile in urine during pregnancy and non-pregnancy periods. (A) positive ions, (B) negative ions. QC samples were tightly clustered, representing our experiment was stable and the obtained data met the conditions for subsequent statistical analyses.


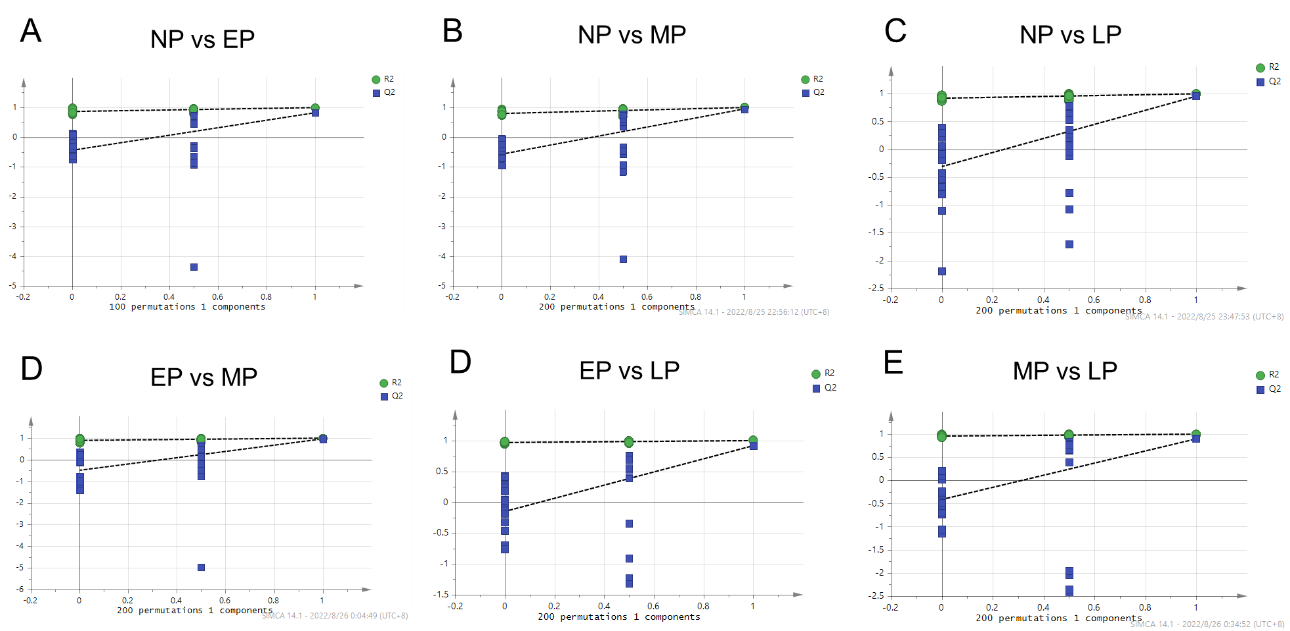


**Figure S2** Permutations plot for partial least squares discriminant analysis (OPLS-DA) models.

The plot above strongly indicates that the original OPLS-DA models are valid. The criteria for validity are: 1) All blue Q2-values to the left are lower than the original points to the right. 2)The blue regression line of the Q2-points intersects the vertical axis (on the left) at, or below zero.
